# Publication and Impact of Preprints Included in the First 100 Editions of the CDC COVID-19 Science Update: Content Analysis

**DOI:** 10.2196/35276

**Published:** 2022-07-15

**Authors:** Jeremy Otridge, Cynthia L Ogden, Kyle T Bernstein, Martha Knuth, Julie Fishman, John T Brooks

**Affiliations:** 1 Centers for Disease Control and Prevention Atlanta, GA United States

**Keywords:** preprints, preprint, publishing, publish, bioRxiv, medRxiv, Centers for Disease Control and Prevention, CDC, preprint server, public health, health information, COVID-19, pandemic, publication, Altmetric attention score, Altmetric, attention score, citation count, citation, science update, decision-making

## Abstract

**Background:**

Preprints are publicly available manuscripts posted to various servers that have not been peer reviewed. Although preprints have existed since 1961, they have gained increased popularity during the COVID-19 pandemic due to the need for immediate, relevant information.

**Objective:**

The aim of this study is to evaluate the publication rate and impact of preprints included in the Centers for Disease Control and Prevention (CDC) COVID-19 Science Update and assess the performance of the COVID-19 Science Update team in selecting impactful preprints.

**Methods:**

All preprints in the first 100 editions (April 1, 2020, to July 30, 2021) of the Science Update were included in the study. Preprints that were not published were categorized as “unpublished preprints.” Preprints that were subsequently published exist in 2 versions (in a peer-reviewed journal and on the original preprint server), which were analyzed separately and referred to as “peer-reviewed preprint” and “original preprint,” respectively. Time to publish was the time interval between the date on which a preprint was first posted and the date on which it was first available as a peer-reviewed article. Impact was quantified by Altmetric Attention Score and citation count for all available manuscripts on August 6, 2021. Preprints were analyzed by publication status, publication rate, preprint server, and time to publication.

**Results:**

Of the 275 preprints included in the CDC COVID-19 Science Update during the study period, most came from three servers: medRxiv (n=201, 73.1%), bioRxiv (n=41, 14.9%), and SSRN (n=25, 9.1%), with 8 (2.9%) coming from other sources. Additionally, 152 (55.3%) were eventually published. The median time to publish was 2.3 (IQR 1.4-3.7). When preprints posted in the last 2.3 months were excluded (to account for the time to publish), the publication rate was 67.8%. Moreover, 76 journals published at least one preprint from the CDC COVID-19 Science Update, and 18 journals published at least three. The median Altmetric Attention Score for unpublished preprints (n=123, 44.7%) was 146 (IQR 22-552) with a median citation count of 2 (IQR 0-8); for original preprints (n=152, 55.2%), these values were 212 (IQR 22-1164) and 14 (IQR 2-40), respectively; for peer-review preprints, these values were 265 (IQR 29-1896) and 19 (IQR 3-101), respectively.

**Conclusions:**

Prior studies of COVID-19 preprints found publication rates between 5.4% and 21.1%. Preprints included in the CDC COVID-19 Science Update were published at a higher rate than overall COVID-19 preprints, and those that were ultimately published were published within months and received higher attention scores than unpublished preprints. These findings indicate that the Science Update process for selecting preprints had a high fidelity in terms of their likelihood to be published and their impact. The incorporation of high-quality preprints into the CDC COVID-19 Science Update improves this activity’s capacity to inform meaningful public health decision-making.

## Introduction

Preprints are publicly available, non-peer reviewed articles posted to various servers such as medRXiv and bioRxiv. Preprints first appeared as information exchange groups in 1961. However, due to resistance from journals, information exchange groups closed in 1967 [1]. In 1991, an automated email server that later became arXiv, a popular modern preprint server, was established [1]. In 2019, Cold Spring Harbor Laboratory collaborated with Yale University to launch medRxiv [1]. Preprints have gained popularity and credibility during the COVID-19 pandemic due to the need for rapid, relevant information to respond to the pandemic. To help inform the public health response to COVID-19, the Centers for Disease Control and Prevention (CDC) created the COVID-19 Science Update [2].

The COVID-19 Science Update provides brief summaries of new COVID-19–related articles on topics such as health equity, vaccines, variants, natural history, and testing, among others [2]. To provide the most relevant and timely information, the COVID-19 Science Update includes both published (peer-reviewed) articles and manuscript preprints. In collaboration with the World Health Organization (WHO), the Stephen B. Thacker CDC Library does a daily systematic (exhaustive, reproducible, and defensible) search for all COVID-19–related articles, which are then cleaned and deduplicated to be included in the WHO COVID-19 Database. The full search strategy for creating this database can be found on the WHO COVID-19 Database website [3]. The resulting articles are sent to the Science Update team within the CDC COVID-19 Response, who then select peer-reviewed articles and preprints on public health priority topics in the CDC Science Agenda for COVID-19 [4] and CDC COVID-19 Response Health Equity Strategy [5].

The COVID-19 Science Update was piloted biweekly beginning in April 2020 and has been publicly available since September 2020 (previous editions were retroactively posted on the internet) [2]. In November 2020, public release became weekly, and an email subscription became available in February 2021. The objective of this analysis is to evaluate the publication rate (percent of preprints published in peer-reviewed journals) and impact (eg, Altmetric Attention Score [6] and citation count) of preprints included in the COVID-19 Science Update and to assess the performance of the COVID-19 Science Update team in selecting impactful preprints.

## Methods

All preprints in the first 100 editions (April 1, 2020, to July 30, 2021) of the COVID-19 Science Update were included in this analysis. Some preprints were eventually published (categorized as “peer-reviewed preprint”) whereas others were not published (categorized as “unpublished preprint”). Time to publish was the time interval between the date on which a preprint was first posted and the date on which it was first available as a peer-reviewed article. Impact was quantified (median and interquartile range) using both Altmetric Attention Score (a weighted measure of attention received from academic, news, and social media sources) and citation count (also from Altmetric) for all available articles (peer reviewed or not) on August 6, 2021. For peer-reviewed preprints, impact metrics only measure impact accumulated following publication. To allow time for preprints to be published and to accumulate impact, we restricted comparisons to items included in the 2020 editions (through Edition 70) of the COVID-19 Science Update. Statistical comparisons (Mood median test) were performed in Minitab with *P*<.05 for statistical significance. Preprints were analyzed by publication status, publication rate, preprint server, and time to publication.

## Results

**Figure 1:**
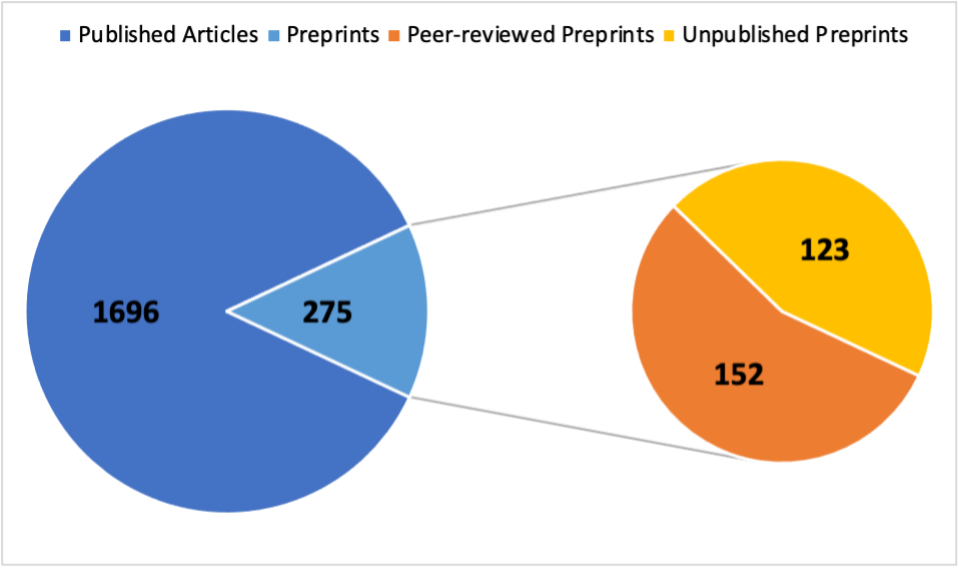
Breakdown of the types of articles included in the first 100 editions (April 2020 to July 2021) of the CDC (Centers for Disease Control and Prevention) COVID-19 Science Update. The left pie displays the overall number of preprints and published articles. The right pie shows how many of the preprints in the left pie were peer reviewed or remained unpublished as of August 6, 2021.

Among the 1,971 articles in the COVID-19 Science Update among the analysis period, 275 (14%) were preprints (Figure 1), most of which came from 1 of the following 3 servers: medRxiv (n=201, 73.1%), bioRxiv (n=41, 14.9%), or Lancet preprints with SSRN (n=25, 9.1%), with 8 (2.9%) coming from other sources. More than half (152/275, 55.3%) were published within the analysis period (April 1, 2020, to August 6, 2021). The median time to publish was 2.3 months (IQR 1.4-3.7). When preprints posted in the last 2.3 months were excluded (to account for the time to publish), the publication rate was 67.8% (143/211). Preprints included in the COVID-19 Science Update were published in 76 different journals, and 18 journals published at least three. Moreover, 2 journals (New England Journal of Medicine and Clinical Infectious Diseases) published 11 preprints each.

Peer-reviewed articles included in the COVID-19 Science Update (n=1696) had a median Altmetric Attention Score of 365 (IQR 64-1316) and median citation count of 33 (IQR 9-111) (Table 1). For peer-reviewed preprints (n=152), median Altmetric Attention Score was 265 (IQR 29-1896) and median citation count was 19 (IQR 3-101). For unpublished preprints (n=123), Median Altmetric Attention Score was 146 (IQR 22-552) and median citation count was 2 (IQR 0-8).

To account for the differences in time that articles have been publicly available, the analytic sample was restricted to only published articles (n=1,140) and preprints (n=73, 50 peer reviewed and 23 unpublished) that were included in the 2020 editions of the COVID-19 Science Update (through edition 70; Table 1). Among the 73 preprints, 50 (68.4%) were published. The median Altmetric Attention Score for published articles (328, IQR 57-1224) was higher than that of unpublished preprints (83, IQR 9-231; *P*=.002). The difference in Altmetric Attention Score between peer-reviewed (260, IQR 43-1817) and unpublished preprints was not statistically significant, likely due to small sample sizes (*P*=.09). The median citation counts for peer-reviewed preprints (55, IQR 9-148) and published articles (43, IQR 15-149) were significantly higher *(P*<.001) than that for unpublished preprints (4, IQR 1-9). There was no significant difference in citation count between peer-reviewed preprints and published articles (*P*=.23).

**Table 1:** Median Altmetric Attention Score and citation count for articles and preprints included in the first 100 editions (April 2020 to July 2021) of the CDC (Centers for Disease Control and Prevention) COVID-19 Science Update, as of August 6, 2021.

Article type				Articles, n (%)	Median Altmetric Attention Score (IQR)	Median citation count, n (IQR)
**Editions 1-100**						
	**Total**			1971	324 (56-1249)	28 (7-93)
		Published		1696 (86)	365 (64-1316)	33 (9-111)
		**Preprints**		275 (14)	174 (22-899)	6 (1-29)
			Peer reviewed^a^	152 (8)	265 (29-1896)	19 (3-101)
			Unpublished	123 (6)	146 (22-552)	2 (0-8)
**Editions 1-70 (2020), to account for time to accumulate impact**						
	**Total**			1213	312 (55-1194)	43 (14-147)
		Published		1140 (94)	328 (57-1224)^b^	43 (15-149)^b^
		**Preprints**		73 (6)	150 (25-866)	15 (4-72)
			Peer reviewed^a^	50 (4)	260 (43-1817)	55 (9-148)^b^
			Unpublished	23 (2)	83 (9-231)	4 (1-9)

^a^Two versions of these papers exist: the original preprint that remains on the preprint server and the peer-reviewed version that is published in a journal

^b^*P*<.05 for difference in Altmetric Attention Score or citation count from unpublished preprints. Testing was only carried out for articles in editions 1-70 to account for the differences in time that articles have had to accumulate impact.

## Discussion

Prior analyses of COVID-19 preprints have found publication rates between 5.7% and 21.1% [7,8]. Preprints included in the COVID-19 Science Update were published at a higher rate than reported elsewhere [7,8], and those that were ultimately published received higher attention scores than unpublished preprints. A high Altmetric Attention Score is indicative of only the total attention a publication receives. It does not differentiate between positive and negative attention, so a high Altmetric Attention Score could equally reflect a publication of high scientific value or one that is widely refuted [6]. Preprints facilitate rapid access to information; however, they are ultimately limited by the absence of peer review and are thus subject to change and may never be published. The ability to discern high-quality impactful preprints is therefore an important tool for public health decision-making. Despite these limitations, the findings of our analysis indicate that the COVID-19 Science Update process for selecting preprints is robust, with high fidelity in terms of the likelihood of preprints to be published and to be impactful. The incorporation of high-quality preprints into the COVID-19 Science Update improves this activity’s capacity to provide timely information, which could have an impact on meaningful public health decision-making.

## Abbreviations

CDC: Centers for Disease Control and Prevention
WHO: World Health Organization

## References

[1] Cobb M (2017). The prehistory of biology preprints: A forgotten experiment from the 1960s. PLoS Biol.

[2] COVID-19 Science Update. Centers for Disease Control and Prevention.

[3] WHO COVID-19 Research Database: user guide and information. World Health Organization.

[4] CDC Public Health Science Agenda for COVID-19. Centers for Disease Control and Prevention.

[5] Health Equity: Promoting Fair Access to Health. Centers for Disease Control and Prevention.

[6] What does Altmetric do?. Altmetric.

[7] Añazco D, Nicolalde B, Espinosa I, Camacho J, Mushtaq M, Gimenez J, Teran E (2021). Publication rate and citation counts for preprints released during the COVID-19 pandemic: the good, the bad and the ugly. PeerJ.

[8] Fraser Nicholas, Brierley Liam, Dey Gautam, Polka Jessica K, Pálfy Máté, Nanni Federico, Coates Jonathon Alexis (2021). The evolving role of preprints in the dissemination of COVID-19 research and their impact on the science communication landscape. PLoS Biol.

